# First Iranian Family with a Novel Missense Variant in *MYO9B* Gene Causing Charcot–Marie–Tooth Disease

**DOI:** 10.34172/aim.33244

**Published:** 2025-04-01

**Authors:** Maryam Beheshtian, Maryam Mozaffarpour Nouri, Fatemeh Ahangari, Mina Makvand, Banafsheh Salmani, Ariana Kariminejad, Hossein Najmabadi, Shahriar Nafissi

**Affiliations:** ^1^Kariminejad-Najmabadi Pathology & Genetics Center, Tehran, Iran; ^2^Genetics Research Center, University of Social Welfare and Rehabilitation Sciences, Tehran, Iran; ^3^Neuromuscular Research Center, Tehran University of Medical Sciences, Tehran, Iran; ^4^Department of Neurology, Shariati Hospital, Tehran University of Medical Sciences, Tehran, Iran

**Keywords:** Charcot–Marie–Tooth disease, Iranian family, Motor and sensory neuropathy, Mutations, MYO9B gene

## Abstract

A heterogeneous clinical and genetic Charcot–Marie–Tooth (CMT) disease, with peripheral nerve damage resulting in chronic motor and sensory polyneuropathy, has been linked to the mutation in over a hundred genes. We report the adult onset of CMT in three siblings of an Iranian family manifesting with muscle weakness and wasting, foot drop, and pes cavus. Whole-exome sequencing (WES) identified a novel homozygous missense mutation in the *MYO9B* gene, inherited from obligatory carrier parents. This likely pathogenic variant contributes to chronic demyelinating sensorimotor polyneuropathy and conduction blocking in the ulnar and median nerves in these patients. To our knowledge, our study is the first report on MYO9B-related CMT in Iranian patients. Previously, a few variations in the *MYO9B* gene were reported to cause CMT. Here we emphasize the potential disruptive role of the detected variant of *MYO9B* in CMT pathogenesis and also highlight the importance of WES for the proper diagnosis of CMT disease. We also compared the clinical presentations of Iranian and Italian patients expanding the clinical and mutational spectrum of MYO9B-related neuropathies.

## Introduction

 Hereditary neuropathies are diverse disorders that can be classified into two main categories: motor sensory neuropathy and sensory autonomic neuropathy (SAN). These disorders are diagnosed by evaluating clinical signs, symptoms, mode of inheritance, and characteristics of electrophysiology study.^1,2^

 Charcot-Marie-Tooth (CMT), with an average prevalence of 4 in 10 000, is a hereditary motor sensory neuropathy. CMT consists of a clinical spectrum from mild muscle weakness to severe and progressive muscle wasting, limb and finger deformities, and sensory loss. This condition can be inherited in three different patterns: autosomal dominant, autosomal recessive, or X-linked.^1-4^

 Mutations in several genes with diverse functions, from myelination and gap junction to axonal structures in peripheral nerves, have been associated with CMT. Duplications of the *PMP22* gene, causing CMT1A, is the most prevalent subtype, while gene deletions, point mutations, and copy number variations have also been reported to cause CMTs.^1,3^

 There are two main subtypes of CMT, namely CMT1 and CMT2, which are distinguished based on neurophysiology and nerve biopsy, specifically related to myelin and axon involvement. However, there are also intermediate forms of CMT that fall between these two subtypes in terms of nerve conduction velocity (NCV), with varying degrees of overlap.^2^

 Genetic testing is a suitable method to directly test for genetic causes when there is supporting evidence within the family. Additionally, it serves as a crucial test to validate electromyography (EMG) and NCV findings. In this sense, we present CMT cases due to a *MYO9B* substitution variant not previously detected in Iranian CMT patients. A written informed consent was obtained from the patient, before publication of this report.

## Case Report

 A 52-year-old female patient, who had typical motor development in infancy and childhood, has experienced difficulty wearing slippers since the age of 32 and then has had multiple instances of ankle sprains. The muscle weakness in the legs gradually deteriorated and extended to the upper limbs. Neurological examination revealed normal mental status and cranial nerves. Proximal upper and lower limb muscles had normal strength but prominent distal upper limb weakness and muscle wasting were observed (finger extensors 4, thumb abduction 2, finger abduction 3). There was bilateral foot drop, wasting of legs and feet muscles, and pes cavus deformity (foot dorsiflexors 3, plantar flexors 4-) (Supplementary file 1, Figure S1B). Deep tendon reflexes were absent in the lower limbs. Pinprick sensation was normal but vibration and position sense were impaired in the feet. She did not exhibit scoliosis, lordosis, or chest wall deformity. Her EMG-NCV results indicated the presence of chronic demyelinating sensorimotor polyneuropathy (Supplementary file 1, Table S1). Conduction blocking was detected in both the bilateral ulnar and left median nerves. Compound muscle action potentials amplitudes and motor nerve conduction velocities were reduced. Sensory nerve action potentials were absent in the hands and feet.

 Both parents were dead, but were not reported to have manifested any sensory or motor disturbances throughout their lives. They were first cousins and had no other relevant family history of a similar disease. The proband has four sisters, two of whom were affected by a similar neurological condition. One of the affected sisters (49 years old) manifested progressive distal lower weakness since 39 years of age, which gradually involved both hands (Supplementary file 1, Figure S1A-III.5). She did not have any sensory symptoms. On neurological examination, mental status and cranial nerves were found normal. No optic atrophy or hearing loss was detected. Arm muscles had normal strength but there was wasting and weakness of hand muscles (finger extensors 5, thumb abduction 4-, finger abduction 3). Proximal lower limb muscles were normal but there was bilateral foot drop and the legs and feet were wasted (foot dorsiflexors 3, plantar flexors 5). Deep tendon reflexes were 2 + in upper limbs but knee and ankle jerks were absent. Pinprick sensation was normal but vibration sense was reduced in both feet. Pes cavus deformity was seen. Nerve conduction studies were compatible with chronic demyelinating sensorimotor polyneuropathy. Conduction blocking was seen in both median and the ulnar motor studies and the motor conduction velocities of ulnar and median were 33 and 39 m/s, respectively. Tibial and peroneal motor studies showed low amplitude with velocities of 18 and 27 m/s, respectively. Sensory potentials were low amplitude in the hands and the surals were unobtainable.

 Based on clinical signs, symptoms, and electrophysiology findings, the diagnosis of CMT was suggested for this index patient (Supplementary file 1, Figure S1A-III.4) and initially referred to the Kariminejad – Najmabadi Pathology & Genetics Center (KNPGC) for genetic screening of deletion or duplication in the *PMP22* gene, on chromosome 17. The MLPA analysis (P033 MRC Holland) for this individual revealed no duplication or deletion of a 1.5 Mb region on chromosome 17p11.2 (CMT/HNPP region).

 Subsequently, the proband’s DNA sample, which was extracted from peripheral blood using the standard salting out method, was subjected to whole-exome sequencing (WES) using Twist Exome V2.0. We captured the patient’s DNA and the NovaSeq 6000 was applied to sequence exonic regions as stated by the manufacturer’s protocols.

 The mean depth of coverage of the exons of the human genome based on CCDS Release 22 was 81X with 97.33% and 96.75% coverage at 10x and 20x, respectively. We identified the c.848G > T variant in the *MYO9B* gene, on chromosome 19 in homozygous state in this proband. The mutant amino acids were located in the motor domain of the *MYO9B* gene, at 146–953. (p.Gly283Val). (Supplementary file 1, Figure S2A).

 This variant was not found in the Genome Aggregation Database (gnomAD). Additionally, we did not find it in the Iranian genomic population database (Iranome).^5^ *In - silico* prediction tools (SIFT, Polyphen2, MutationTaster, MutationAssessor, FATHMM, FATHMM MKL) were in support of the probable pathogenicity of this substitution and then it was predicted that this variant is deleterious and disease-causing. This missense variant occurs at a highly conserved genomic sequence, in the motor domain of the protein encoded by the *MYO9B* gene (Supplementary file 1, Figure S2A-C). According to the American College of Medical Genetics and Genomics (ACMG) guidelines, the c.848G > T; p.Gly283Val (NM_004145.4: chr19:17256214G > T) variant was classified as likely pathogenic (PM1 + PM2 + PP1 + PP3 + ).^6^

 We designed a specific set of primer pairs for PCR amplification (342 bp product long; forward: aatataaaagcacagtgtaaaatgaga, reverse: ccttcacctcaaagacctaaaa). The PCR products were directly sequenced on the automated genetic analyzer (Applied Biosystems 3130xl; USA) (Supplementary file 1, Figure S1C). The sequences were compared with normal sequences using Codon Code Aligner (version 8.0.1). Direct Sanger sequencing confirmed the presence of this missense variant (p.Gly283Val) in the other affected family members (Supplementary file 1, Figure S1A-III.5).

 To our knowledge, this variant has not been previously reported elsewhere. Our WES did not reveal other plausible, causative candidate variants in the proband’s DNA sample. Thereafter, this novel missense substitution variant in exon 3 of the *MYO9B* gene (c.848G > T) indicates that this variation is associated with CMT.

## Discussion

 CMT is a genetically and clinically heterogeneous disease classified as motor and sensory peripheral neuropathies.^7^ Here, we present a novel variant in the *MYO9B* gene that causes CMT disease in Iranian patients. To our knowledge, this is the first study to detect a variation in *MYO9B* causing CMT in an Iranian family. A few *MYO9B* variations have been previously reported to be associated with CMT disease, including in the study by Cipriani et alwho described novel variants located in structural domains of this gene in two unrelated Italian families that manifested HMN/CMT2.^8^

 MYO9B is a myosin motor protein which has signaling properties and rho-GTPase activity.^9,10^ The protein encoded by the *MYO9B* gene is an unconventional myosin family of actin-based motor molecules converting chemical energy that comes from hydrolysis of adenosine triphosphate (ATP) into mechanical power.^10^ It contains the motor head region, four IQ motifs and a RhoGAP domain located at the C- terminus. IQ motifs bind the Ca^2+^ sensor calmodulin, generating an arm which is involved in motor movements. Evidence indicates that a RhoGAP domain plays a role in deactivating RhoA-signaling by accelerating the hydrolysis of guanosine triphosphate (GTP). Furthermore, research has revealed that the RhoGAP domain interacts with the cytosolic region of the single transmembrane receptor Robo1, inhibiting the guanosine triphosphatase (GTPase)-activating protein (GAP) activity.^9^ With a single-headed structure of myosin, this complex protein displays processive actin-based movement.^11,12^

 In 2005, celiac disease, an immune-related disorder, was reported to be associated with a common variant located in intron 28 of the *MYO9B* gene.^13^ In 2022, Cipriani et al showed a possible alteration of MYO9B localization and signaling activity due to motor domain change from Tyr176His in one of two Italian families with CMT2. Their findings suggest that motor activity and protein expression levels were impaired due to the occurrence of an amino acid substitution, from tyrosine to histidine, at codon 176 in the MYO9B motor domain (Supplementary file 1, Figure S2A). Additionally, they found compound heterozygous variants in another affected family, located in the Ras association domain without elucidated function. The patients showed similar signs and symptoms, including disease onset at under 20 years of age and slow progression from a moderate to severe phenotype in adulthood. They had abnormal motor strength, motor function, and vibration, indicating a loss of myelinated axons. Sensory–motor polyneuropathy with similar features was shown in an electrophysiological examination of the four affected patients.^8^

 Two patients in our study showed distal leg muscle weakness, which gradually extended to the upper limbs. Hand muscle weakness and wasting were prominent. The EMG-NCV findings suggested chronic demyelinating sensorimotor polyneuropathy. Conduction blocking was seen in the ulnar and median nerves of both affected siblings. In the WES analysis of 509 neuromuscular genes, we detected p.Gly283Val, a semiconservative amino acid substitution, in homozygous status, that was classified as likely pathogenic.^6^ It may affect the secondary structure of the protein and alter some properties, indicating that it might be located at an important functional domain in *MYO9B*. However, further functional investigation is needed to clarify the role of this variation in this gene that causes CMT disease.

 Two Iranian and four Italian patients with CMT attributed to the variations in *MYO9B* gene have shared clinical manifestations as follows: atrophy and weakness of distal muscles in the upper and lower limbs, gait disturbance, onset with foot dorsiflexor weakness, somatic sensory dysfunction, and reduced to absent knee and ankle deep tendon reflexes. In the EMG-NCV study, all patients showed sensorimotor polyneuropathy. However, two Italian patients had conduction slowing in the axonal range (38-46 m/s), and the other two had it in the demyelinating range (32-33 m/s in ulnar nerves). The authors interpreted these electrodiagnostic findings as indicating slowing in the “intermediate” range.^8^ Both of our patients had clear demyelinating features in the electrodiagnostic tests.

 Sensory dysfunction in the legs that manifested in the late teens was found in one of the six patients. Postural instability was also reported in an Italian patient. In one of the two siblings in an Italian family, hemifacial microsomia, congenital unilateral hearing loss, and nerve fiber thinning in the left eye suggestive of damage to the optic nerve were noted.^8^ This may be indicative of interfamilial clinical phenotype variability, even in one family with the same variation (Supplementary file 1, Table S1).

 The involvement of intrinsic muscles in the hands was reported in all Italian patients (Supplementary file 1, Table S1). None of the patients showed motor delay, weakness of facial musculature or abnormality of the vertebral column.

## Conclusion

 Our study reinforces the importance of next-generation sequencing in providing accurate and fast genomic data for proper clinical CMT disease diagnosis. It will also help in affirming the importance of the MYO9B gene in causing CMT disease. Our study of families from Iran and the comparison with previous reports of Italian families also expands the spectrum of the phenotype and genotype causing CMT by identifying novel variants in the MYO9B gene.

## Supplementary Files

Supplementary file 1 contains Figure S1 (Family pedigree, Deformity of foot, and Electropherograms), Figure S2 (Schematic representation of domains of protein, Genomic region of MYO9B gene, and Evidence of pathogenicity) and Table S1 (Genetic, clinical, and electrophysiological data).
