# Supplementary material for: First Iranian Family with a Novel Missense Variant in MYO9B Gene Causing Charcot–Marie–Tooth Disease
Source: Arch Iran Med. 2025 Apr 1;28(4):236–9. doi: 10.34172/aim.33244 (PMC12085794; doi:10.34172/aim.33244)
Supplement: Supplementary file 1 — contains Figure S1 (Family pedigree, Deformity of foot, and Electropherograms), Figure S2 (Schematic representation of domains of protein, Genomic region of MYO9B gene, and Evidence of pathogenicity) and Table S1 (Genetic, clinical, and electrophysiological data). [file aim-28-236-s001.pdf]

Arch Iran Med. April 2025;28(4):236-S1

Doi: 10.34172/aim.33244

Supplementary file 1

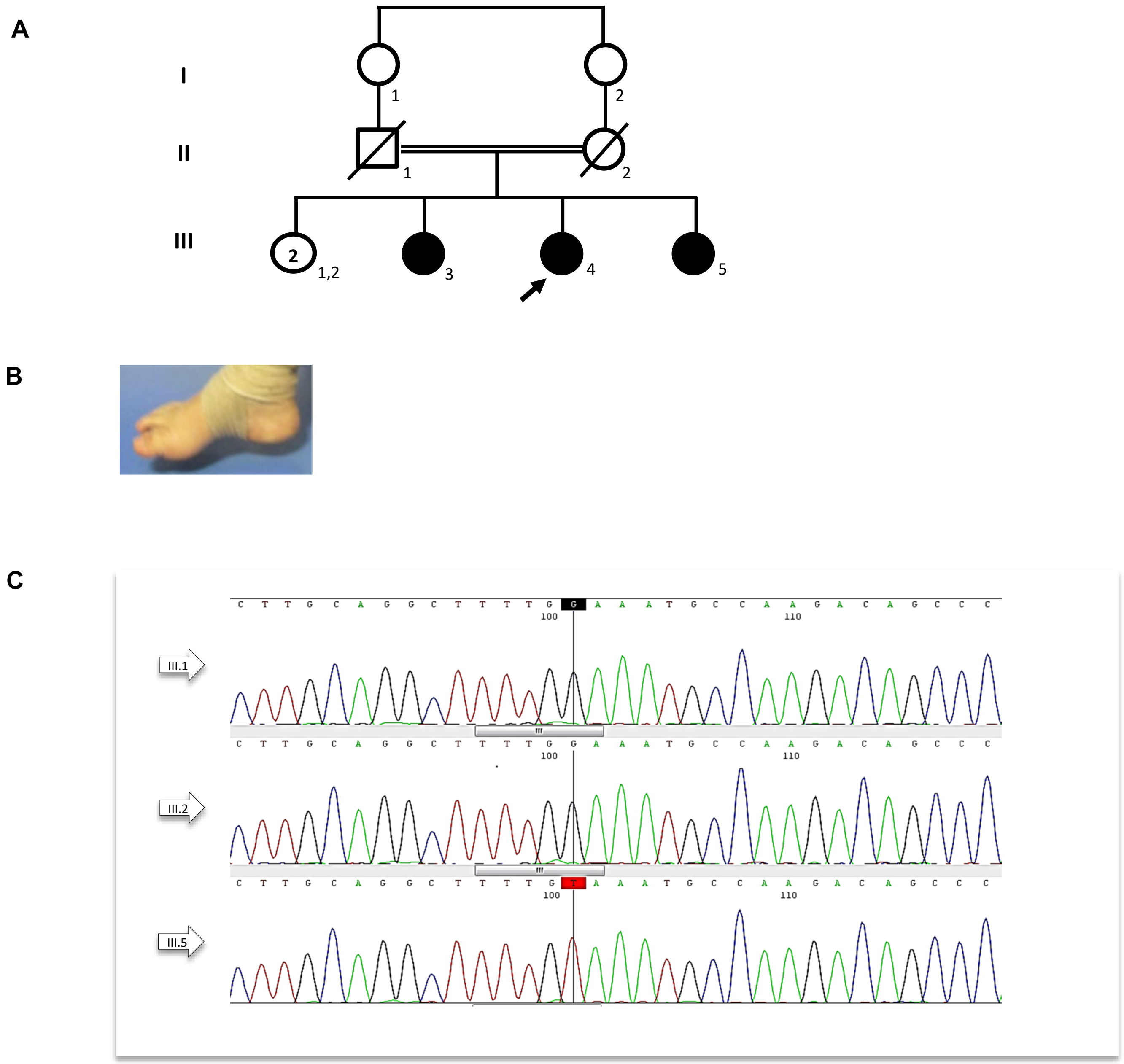

Figure S1. A) Family pedigree with autosomal recessive CMT carrying a novel homozygous variant c.G848T in the *MYO9B* gene (resulting in amino acid substitution p.Gly283Val). The black arrow shows the proband (III.4). The affected sister (III.5) was a homozygote carrying the recessive mutant alleles in the *MYO9B* gene. The other affected sibling (III.3) was not screened for this mutation. B) Deformity of the high arch of the proband's right foot. C) Electropherograms of affected and healthy individuals of this family.

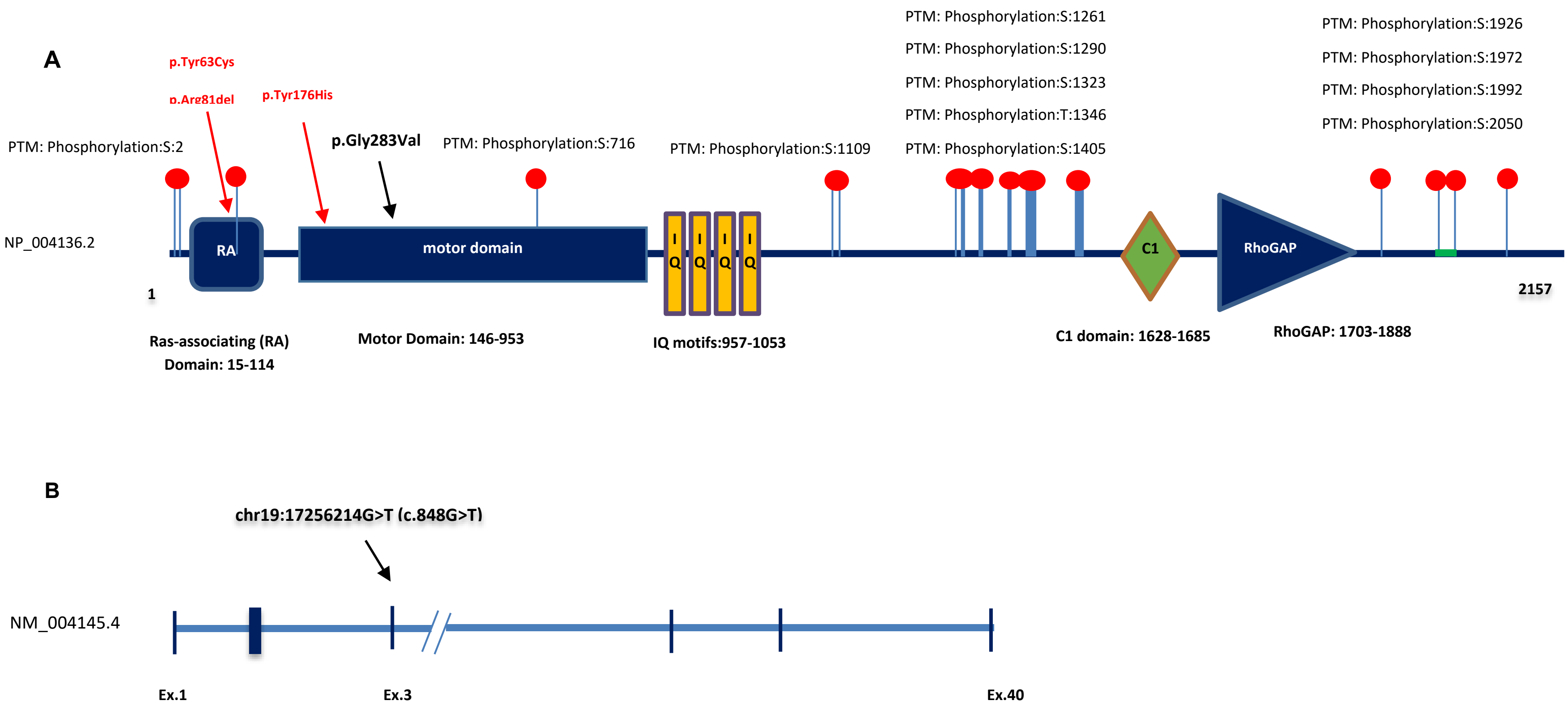
A
p.Tyr63Cys
p.Arg81del
p.Tyr176His
p.Gly283Val
PTM: Phosphorylation:S:2
PTM: Phosphorylation:S:716
PTM: Phosphorylation:S:1109
PTM: Phosphorylation:S:1261
PTM: Phosphorylation:S:1290
PTM: Phosphorylation:S:1323
PTM: Phosphorylation:T:1346
PTM: Phosphorylation:S:1405
PTM: Phosphorylation:S:1926
PTM: Phosphorylation:S:1972
PTM: Phosphorylation:S:1992
PTM: Phosphorylation:S:2050
NP_004136.2
RA
motor domain
IQ
C1
RhoGAP
1
2157
Ras-associating (RA) Domain: 15-114
Motor Domain: 146-953
IQ motifs:957-1053
C1 domain: 1628-1685
RhoGAP: 1703-1888
B
chr19:17256214G>T (c.848G>T)
NM_004145.4
Ex.1
Ex.3
Ex.40

C

Species	Conservation: Nucleotide level change							
	PhyloP				PhastCons			
	5.455				1			
Human	**T**	**T**	**T**	**G**	**G**	**A**	**A**	**A**
Chimp	**t**	**t**	**t**	**g**	**G**	**a**	**a**	**a**
Rhesus	t	t	t	g	**G**	a	a	a
Mouse	t	t	t	g	**G**	g	a	a
Baboon	t	t	t	g	**G**	a	a	a
Caw	t	t	t	g	**G**	a	a	a
Opossum	t	t	t	g	**G**	a	a	a
Chicken	t	t	t	g	**G**	a	a	a
Zebrafish	t	t	t	g	**G**	c	a	a
**Population database**	Variant was neither found in gnomAD nor 1000G.							
**Iranian population database (iranome.ir)**	Not found							
**In-silico prediction**								
MutationTaster	Disease causing							
SIFT Pred (C)	Damaging							
Polyphen2 HVAR Pred (C)	Probably damaging							
MutationAssessor	Predicted functional (high)							
FATHMM	Damaging							
FATHMM MKL Coding	Damaging							
CADD score	25.1							

Figure S2. Schematic Representation of Domains of Protein (A) encoded by the *MYO9B* gene and the genomic (B) region of this gene[1,2] (C) evidence in favor of pathogenicity.

The black arrow shows the location of the variant identified in the proband and affected sibling in this study. The red arrows display additional variants in this gene detected by Cipriani *et al.*, 2022. Supporting evidence for the pathogenic role of the detected variant in this study is presented in C. PTM: post-translational modifications (PTMs) and/or processing events.

[1] *Uniprot. Available from https://www.uniprot.org/uniprotkb/Q13459/entry#structure. Accessed March 2024.*

[2] *NCBI. Available from https://www.ncbi.nlm.nih.gov/protein/NP_004136.2?report=graph.Accesseed March 2024.*

Table S1. Genetic, Clinical, and Electrophysiological Data from the Affected Siblings and Four Additional Patients with CMT Disease Type 2 Neuropathies, as Reported by Cipriani *et al.* (2023)[1].

Data	**Patient, III,4 (Female) (this study)**	**Patient, III,5 (Female) (this study)**	**Female (Pedigree A, II,1; PMID: 36260368)**	**Male (Pedigree A, II,2; PMID: 36260368)**	**Female (Pedigree B, II,1; PMID: 36260368)**	**Female (Pedigree B, II,2; PMID: 36260368)**
**Gene alteration (Zygosity)**	c.848G>T (homozygous state)	c.848G>T (homozygous state)	c.526T>C (homozygous state)	c.526T>C (homozygous state)	c.188A>G & c.241_243del (compound heterozygous state)	c.188A>G & c.241_243del (compound heterozygous state)
**Age of onset**	Adulthood	Adulthood	Childhood	Adolescence	Childhood to teen	Late teens
**Onset symptom**	Distal lower limb weakness	Distal lower limb weakness	Difficulty walking	Difficulty walking	Postural instability & then difficulty walking	_
**Motor delay** (HP:0001270)	-	-	ND	ND	ND	ND
**Weakness of facial musculature** (HP:0030319)	-	-	ND	ND	ND	ND
**Limb muscle weakness** (HP:0003690)	+ (Distal upper & lower)	+ (Distal upper & lower)	+	+	+	+
**Gait disturbance** (HP:0001288) or **Difficulty walking** (HP:0002355)	+	+	+	+	+	+
**Foot dorsiflexor weakness** (HP:0009027)	+ (Foot drop, bilateral)	+ (Foot drop, bilateral)	+ (Foot drop, bilateral)	+ (Foot drop, bilateral)	+ (Foot drop, bilateral)	+ (Foot drop, bilateral)
**Impaired toe-walking ability** (HP:0034052)	+	-	+	-	+	+
**Somatic sensory dysfunction** (HP:0003474)	Reduced vibration sensation (feet)	Reduced vibration sensation (feet)	Reduced vibration sensation (ankle)	Reduced vibration sensation (knee)	Absent vibration sensation (knees)	Reduced to absent vibration sensation (knees)
**Abnormality of the vertebral column** (HP:0000925)	-	-	ND	ND	ND	ND

**Deep tendon reflex**	Knee and ankle absent	Knee and ankle absent	Knee reduced/ ankle absent	Knee reduced/ ankle absent	Knee & biceps reduced/ ankle absent	Knee & biceps reduced/ ankle absent
**Upper limbs strength**	Finger extensor 4, Finger abduction 3, Thumb abduction 2	Finger extensors 5, Finger abduction 3, Thumb abduction 4-	Thenar> hypothenar, hand	Thenar> hypothenar, hand	Wrist extensor 4+, Finger extensor 4, Intrinsic hand muscles ≤2	Triceps 4+, Wrist extensor 4, Finger extensor 4-, Intrinsic hand muscles ≤3/5
**Lower limbs strength**	Ankle dorsiflexors 3 Plantar flexors 4-	Ankle dorsiflexors 3 Plantar flexors 5	Ankle dorsiflexor > plantar flexor	Ankle dorsiflexor	Ankle dorsi- and plantar flexion ≤2/5	Hamstring 4, Ankle dorsi- and plantar flexion ≤2
**Additional clinical findings**	Pes cavus; Difficulty wearing slippers; Transient ankle sprain; Muscle atrophy in hands, legs and feet	Pes cavus; Muscle atrophy in hands, legs and feet	Pes cavus; Muscle atrophy in distal muscle of upper and lower limbs	Muscle cramps in the lower limbs at rest and after exercise; Calf muscle hypertrophy; Muscle atrophy in hands and ankle dorsiflexor muscles	Clawing fingers; High arches; Hammer toes; Muscle atrophy in hands and lower legs	Reduced pinprick sensation (knees); Feet swelling and pain while standing; atrophy in hands and lower legs; High arches; Hammer toes; Hemifacial microsomia; Unilateral congenital hearing loss; Unilateral thinning of the nerve fiber in eye (optic nerve damage)
**Nerve conduction study**						
**Median nerve, SNCV, m/s**	NR	40	32.6	NR	NR	NR
**Median nerve, dCMAP, mV**	1.0	6.6	NR	2.7	0.1	NR
**Median nerve, MNCV, m/s**	35	40	NA	38	24	NR
**Ulnar nerve, SNCV, m/s**	NR	38	ND	NA	NR	NR
**Ulnar nerve, dCMAP, mV**	3.4	5.9	2.2	4.4	2.8	2.9
**Ulnar nerve, MNCV, m/s**	42	34	46	39	33	32
**Tibial nerve, dCMAP, mV**	1.1	1.8	0.1	ND	ND	ND
**Tibial nerve, MNCV, m/s**	31	24	35	ND	ND	ND
**Peroneal nerve, dCMAP, mV**	NR	0.7	NR	1.3	ND	ND
**Peroneal nerve, MNCV, m/s**	NR	28	NA	37	ND	ND

**Sural nerve, SNCV, m/s**	NR	NR	NA	NA		
**Radial nerve, SNCV, m/s**	NR	42	NA	NA	46	NR
**EMG-NCV impression**	demyelinating sensorimotor polyneuropathy	demyelinating sensorimotor polyneuropathy	Axonal sensorimotor polyneuropathy	Axonal sensorimotor polyneuropathy	Demyelinating sensorimotor polyneuropathy	Demyelinating sensorimotor polyneuropathy
**CMTNS[2]**	14	7	16	17	20	31

CMTNS: Charcot–Marie–Tooth Neuropathy Score, Dcmap: distal compound muscle action potential, DML: distal motor latency, MNCV: motor nerve conduction velocity, NA: not applicable, ND: not determined, NR: no response, R: right, SAP: sensory action potential, SNCV: sensory nerve conduction velocity, m/s: meters per second.

[1]*Cipriani S, Guerrero-Valero M, Tozza S, Zhao E, Vollmer V, Beijer D, et al. Mutations in MYO9B are associated with Charcot-Marie-Tooth disease type 2 neuropathies and isolated optic atrophy. Eur J Neurol. 2023;30(2):511-526. doi: 10.1111/ene.15601.*

[2]*Murphy SM, Herrmann DN, McDermott MP, Scherer SS, Shy ME, Reilly MM, et al. Reliability of the CMT neuropathy score (second version) in Charcot-Marie-Tooth disease. J Peripher Nerv Syst. 2011;16(3):191-8. doi: 10.1111/j.1529-8027.2011.00350.x.*
